# DYADIC DISCORDANCE ABOUT DAILY LIVING CHALLENGES AMONG PERSONS WITH COGNITIVE IMPAIRMENT

**DOI:** 10.1093/geroni/igae098.4168

**Published:** 2024-12-31

**Authors:** Ana Sophia Garces Torres, E-Shien Chang, Jerad Moxley, Walter Boot, Wendy Rogers, Sara Czaja, Neil Charness

**Affiliations:** Weill Cornell Medicine/Center on Aging and Behavioral Research, New York, New York, United States; Weill Cornell Medical College, New York, New York, United States; Weill Cornell Medicine, New York, New York, United States; Weill Cornell Medicine, New York, New York, United States; University of Illinois Urbana-Champaign, Champaign, Illinois, United States; Weill Cornell Medicine/Center on Aging and Behavioral Research, New York, New York, United States; Florida State University, Tallahassee, Florida, United States

## Abstract

As the prevalence of older adults with a cognitive impairment (PwCI) continues to increase, it is critical to identify cognitive tasks which are challenging for them, as well as areas of needed support for both the PwCI and their care partner (CP). Identifying discordant reports about daily challenges among dyads may also flag risks for potentially adverse caregiving experiences. This presentation will report on sources of dyadic discordance about daily cognitive tasks. Data were drawn from baseline quantitative and qualitative data from an ongoing, longitudinal mixed methods needs-assessment study called Everyday Needs Assessment for Cognitive Tasks (ENACT). PwCI and their CPs (n=22 dyads) rated the difficulty level that the PwCI experienced across five daily activity categories. The average ages of PwCI and CPs were 74.2 and 64.7 years respectively. Dyads were predominantly spouses/partners (64.7%). Both PwCIs and CPs identified health-related activities (e.g., health information activities, appointment activities, health management activities) as the most challenging. Additionally, the findings indicate that dyadic congruence regarding activity challenges varies according to activity. There was agreement between the PwCI and CPs regarding transportation, r(20)=0.53, p< 0.05 and domestic life, r(20)=0.72, p< 0.01 activities. Despite significant correlation in leisure and recreation activities, r(20)=0.71, p< 0.01, CPs underestimated PwCI’s experienced difficulty, t(21) = 2.70, p< 0.05. Additional qualitative thematic analyses revealed the dynamic role of technology in navigating cognitive challenges. Improved understanding of the areas of dyadic discordance can assist in guiding support interventions for individuals experiencing cognitive challenges and their CP.

